# Fate of Diffusion Restricted Lesions in Acute Intracerebral Hemorrhage

**DOI:** 10.1371/journal.pone.0105970

**Published:** 2014-08-28

**Authors:** Yuan-Hsiung Tsai, Ming-Hsueh Lee, Hsu-Huei Weng, Sheng-Wei Chang, Jen-Tsung Yang, Yen-Chu Huang

**Affiliations:** 1 Department of Diagnostic Radiology, Chang Gung Memorial Hospital at Chiayi, Chang-Gung University College of Medicine, Taoyuan, Taiwan; 2 Department of Neurosurgery, Chang Gung Memorial Hospital at Chiayi, Chang-Gung University College of Medicine, Taoyuan, Taiwan; 3 Department of Neurology, Chang Gung Memorial Hospital at Chiayi, Chang-Gung University College of Medicine, Taoyuan, Taiwan; National Yang-Ming University, Taiwan

## Abstract

**Background:**

Diffusion-restricted lesions on diffusion-weighted imaging (DWI) are detected in patients with intracerebral hemorrhage (ICH). In this study, we aimed to determine the fate of DWI lesions in ICH patients and whether the presence of DWI lesions is associated with functional outcome in patients with ICH.

**Methods:**

This prospective study enrolled 153 patients with acute ICH. Baseline MRI scans were performed within 2 weeks after ICH to detect DWI lesions and imaging markers for small vessel disease (SVD). Follow-up MRI scans were performed at 3 months after ICH to assess the fate of the DWI lesions. We analyzed the associations between the characteristics of DWI lesions with clinical features and functional outcome.

**Results:**

Seventeen of the 153 patients (11.1%) had a total of 25 DWI lesions. Factors associated with DWI lesions were high initial systolic and mean arterial blood pressure (MAP) at the emergency room, additional lowering of MAP within 24 hours, and the presence of white matter hyperintensity and cerebral microbleeds. Thirteen of the 25 DWI lesions (52%) were not visible on follow-up T2-weighted or fluid-attenuated inversion recovery images and were associated with high apparent diffusion coefficient value and a sharper decease in MAP. The regression of DWI lesions was associated with good functional outcome.

**Conclusions:**

More than half of the DWI lesions in the ICH patients did not transition to visible, long-term infarction. Only if the DWI lesion finally transitioned to final infarction was a poor functional outcome predicted. A DWI lesion may be regarded as an ischemic change of SVD and does not always indicate certain cerebral infarction or permanent tissue injury.

## Introduction

Primary intracerebral hemorrhage (ICH) is associated with higher mortality rates and more severe neurological deficits than any other subtype of stroke. [1] Prior studies have found that lesions with restricted diffusion on diffusion-weighted imaging (DWI) were observed in 13–41% of patients with acute ICH. [2]–[7] The DWI lesions were found to be associated with MRI markers of small vessel diseases (SVD) such as cerebral microbleeds (CMB) [3]–[5], [7], [8] and white matter hyperintensity (WMH). [3], [5], [7] Three recent studies found an association between rapid blood pressure reduction and DWI lesions in patients with acute ICH [4], [6], [7].

Although the underlying physiology as well as potential impact on clinical management and outcomes of DWI lesions are not fully understood, DWI lesions have been generally considered as ischemic infarcts in prior studies. [9] Garg et al. [6] found that DWI lesions after ICH are associated with greater acute blood pressure reduction as well as disability and death at 3 months. In another recent study by Kang et al., [5] it was demonstrated that DWI lesions may predict future ischemic stroke, recurrent ICH, and vascular death. Based on these findings, the benefit of acute blood pressure reduction, as suggested by American Heart Association guidelines and some clinical trials, [10], [11] has been suspected of being reduced by the induction of new ischemic lesions observed on DWI. [4], [6], [9] However, whether the presence of a DWI lesion in ICH patients is related to poor functional outcome is still controversial [5].

We therefore sought to determine the fate of DWI lesions and their impact on further functional outcome in patients with ICH. Since DWI lesions may be a marker of vasculopathy, we hypothesized that not all DWI lesions develop into ischemic infarcts and lead to poor functional outcome.

## Methods

### Patients

We performed a prospective longitudinal study of lesions on DWI and apparent diffusion coefficient (ADC) magnetic resonance imaging (MRI) sequences in 153 patients hospitalized with acute spontaneous ICH within 24 hours of symptom onset. Patients were enrolled after their ICH was confirmed by computed tomography (CT). Patients with contraindications to MRI and those who refused to consent to participate in this study were excluded. The study was part of an integrated stroke project at Chang Gung Memorial Hospital and was approved by the Institutional Review Board of Chang Gung Memorial Hospital. All patients, or their families, gave their written informed consent prior to participation in the study. The authors confirm that all data underlying the findings are fully available without restriction.

### MRI acquisition and analysis

All data were collected using a 3 Tesla Siemens Verio MRI system (Siemens Medical System, Erlangen, Germany) with a 32-channel head coil. Patients were scanned at baseline (mean: 3.9 days; range: 1–14 days after ICH) to detect DWI lesions and again at around 3 months after ICH for follow-up. Standard sequences included axial T2*-weighted gradient echo images (repetition time [TR]/echo time [TE] = 600/17.8 ms, excitations = 1, flip angle = 20 degrees, section thickness = 4.0 mm with a gap = 1.2 mm and matrix size = 256×146); axial fluid-attenuated inversion recovery (FLAIR) images ([TR]/[TE] = 9000/85 ms, inversion time = 2500 ms, matrix size 165×256, using the same section thickness) and an axial T2-weighted turbo spin echo images ([TR]/[TE] = 3000/86 ms, matrix size 194×256, using the same section thickness). DWI imaging was performed using an EPI sequence with b = 0 and b = 1000 s/mm^2^, in three dimensions in space and resulting in four images per section. The imaging parameters used for this were TR/TE = 5600/93 ms, matrix size of 130×130, 4.0 mm slice thickness, and a 230-mm field of view (FOV). The apparent diffusion coefficient (ADC) map was derived directly from these diffusion-weighted images.

**Figure 1:**
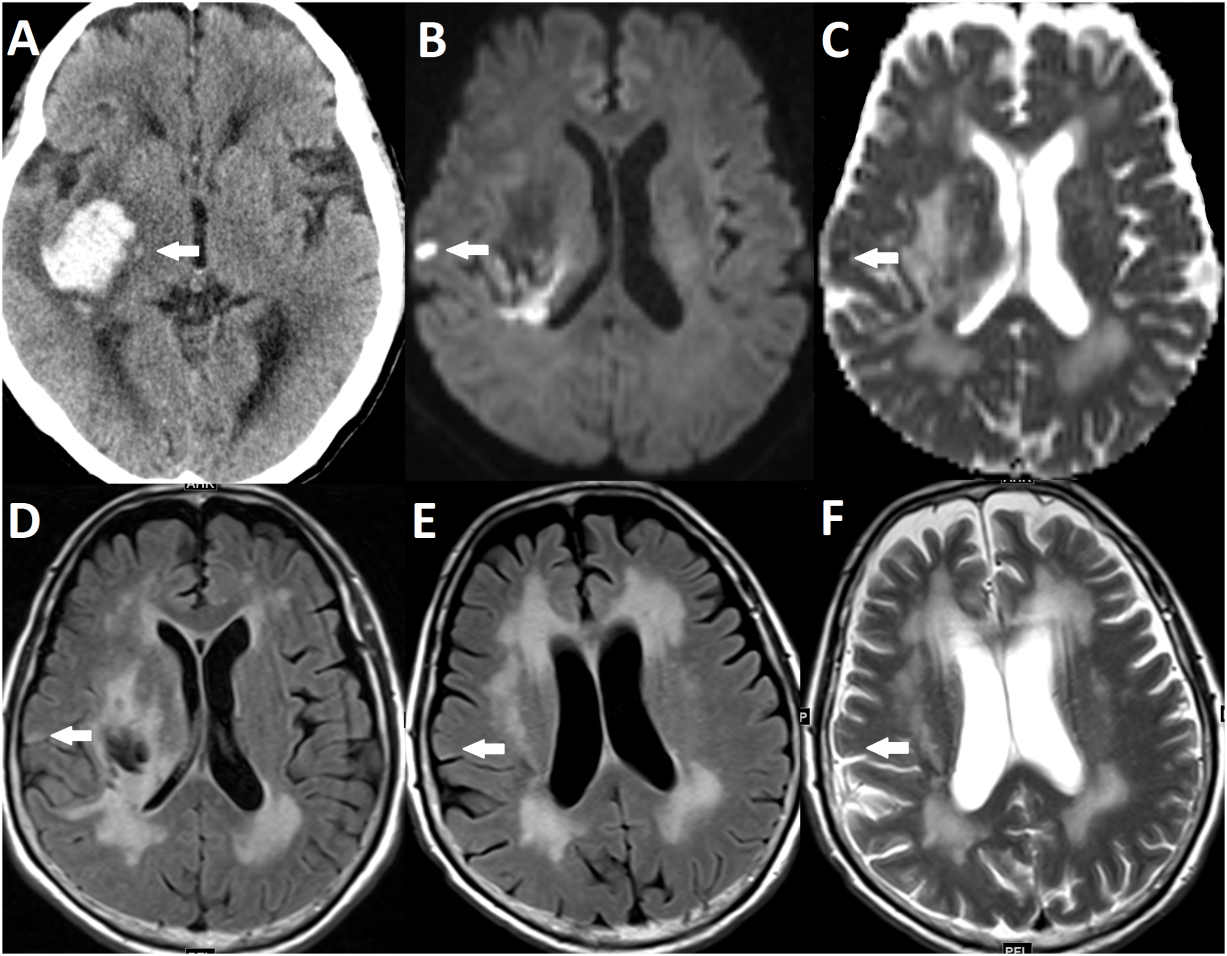
Example of a DWI lesion not visible on follow-up MRI. (A) CT showing ICH in right putamen (arrow). (B, C and D) Baseline MRI showing a DWI lesion that has high signal intensity on DWI (B), low signal intensity on ADC (C) and equivocal high to intermediate signal intensity on FLAIR (D). (E and F) The DWI lesion is not visible on FLAIR (E) and T2WI (F) MRI 3 months after ICH.

A DWI lesion was defined as a high-signal intensity lesion on DWI, accompanying low-signal intensity on ADC. The high-signal intensity lesions in the area of hematoma and surrounding tissue were excluded. The size of a DWI lesion was defined as the greatest diameter of the DWI lesion. The mean ADC value of the DWI lesion was also measured for quantitative analysis. Four MRI markers of SVD, including WMH, enlarged perivascular space (EPVS), lacunae and CMB, have been previously described. [12], [13] We further categorized these lesions according to their anatomic locations because the locations for these lesions may represent different pathophysiologies (Table 1). [13] Since 11 subjects did not have T2*-weighted gradient echo images included in their baseline MRI, the number of subjects for analyzing the association between CMB and DWI lesion was 142. All neuroimaging data were reviewed by an experienced stroke neurologist (Y.C.H.) and a neuroradiologist (Y.H.T.) blinded to clinical information. An illustrated case of an ICH patient with a DWI lesion is shown in Figure 1.

**Table 1:** Demographic and clinical characteristics of patients with and without DWI lesions.

	DWI Positive (n = 17)	DWI Negative (n = 136)	*p*
Age, y	69.2 (12.5)	63.1 (12.9)	0.068
Male gender	8 (47.1)	86 (63.2)	0.196
Risk factors			
Hypertension	17 (100.0)	122 (89.7)	0.165
Diabetes	4 (23.5)	28 (20.1)	0.756
Smoking	4 (23.5)	45 (33.0)	0.584
Alcoholism	3 (17.6)	38 (27.9)	0.562
Previous ischemic stroke or TIA	1 (5.9)	16 (11.8)	0.695
Initial clinical and laboratory data			
Platelet count, 1000/µl	195.2 (56.2)	210.9 (76.3)	0.421
Hemoglobin, g/dl	13.6 (1.5)	14.0 (1.7)	0.383
Creatinine, mg/dl	0.8 (0.2)	1.1 (1.1)	0.331
NIHSS score	14.4 (14.3)	9.4 (9.0)	0.176
GCS score	12.2 (3.4)	13.1 (2.9)	0.239
Blood pressure, mmHg			
SBP at ER	201.9 (28.8)	186.2 (31.0)	0.049*
DBP at ER	117.4 (23.4)	107.6 (22.5)	0.097
MAP at ER	145.5 (23.0)	133.8 (22.2)	0.043*
Lowest SBP within 24 h	137.2 (26.2)	135.8 (19.5)	0.793
Lowest DBP within 24 h	77.3 (17.5)	79.0 (17.9)	0.706
Lowest MAP within 24 h	97.3 (18.9)	98.0 (16.5)	0.872
Delta SBP (ER - lowest within 24 h)	64.7 (28.4)	50.4 (31.0)	0.072
Delta DBP (ER - lowest within 24 h)	40.6 (27.1)	28.6 (22.1)	0.052
Delta MAP (ER - lowest within 24 h)	48.3 (25.7)	35.9 (21.5)	0.030*
Outcomes			
7-day GCS score	12.4 (3.2)	13.3 (2.4)	0.272
3-month mRS 0–2	5 (29.4)	65 (47.8)	0.151
MRI markers of small vessel disease			
WMH, overall	16 (94.1)	96 (70.1)	0.043*
WMH, periventricular	15 (88.2)	95 (69.9)	0.154
WMH, basal ganglia	16 (94.1)	60 (44.1)	<0.001*
EPVS, overall	17 (100.0)	117 (86.0)	0.132
EPVS, centrum semiovale	13 (76.5)	88 (64.7)	0.422
EPVS, basal ganglia	17 (100.0)	115 (84.6)	0.131
Lacune infarct, overall	3 (17.6)	40 (29.4)	0.400
Lacune infarct, cortical	2 (11.8)	25 (18.4)	0.739
Lacune infarct, deep	1 (5.9)	17 (12.5)	0.695
Lacune infarct, brain stem	0 (0.0)	2 (1.5)	1.000
CMB^a^, overall	13 (81.2)	61 (48.4)	0.016*
CMB^a^, lobar	12 (75.0)	29 (23.0)	<0.001*
CMB^a^, deep	12 (75.0)	57 (45.2)	0.033*

a CMB data were unavailable in 11 patients. The effective subject number for CMB analysis is n = 16 for DWI negative; n = 126 for DWI positive.

*Significant differences were defined as those with *p*<0.05.

Results are expressed as number (column %) for nominal variable and mean (SD) for continuous variables.

Abbreviations: DWI = diffusion-weighted imaging; TIA = transient ischemic attack; NIHSS = National Institutes of Health Stroke Scale; GCS = Glasgow Coma Scale; SBP = systolic blood pressure; DBP = diastolic blood pressure; MAP = mean arterial pressure; ER = emergency room; WMH: white matter hyperintensity; EPVS = enlarged perivascular spaces; CMB = cerebral microbleeds; mRS = modified Rankin Scale.

### Clinical assessments

Demographic data (age, gender, time from symptom onset to baseline MRI), presence of vascular risk factors (hypertension, diabetes, smoking, alcoholism and previous ischemic stroke or transient ischemic attack), blood pressure (initial blood pressure measured at the emergency room [ER] and lowest within 24 hours), as well as other laboratory and clinical assessments (platelet count, hemoglobin, creatinine, National Institutes of Health Stroke Scale [NIHSS] scores at the ER, Glasgow Coma Scale [GCS] scores at the ER) were collected for all patients. Patient outcomes, including GCS score at day 7 for acute stage and modified Rankin Scale (mRS) score at 3 months for long-term functional outcome, were also estimated. An mRS of 0–2 was defined as good functional outcome.

### Statistical analysis

All statistical analyses were performed using the Statistical Program for Social Sciences (SPSS) statistical software (version 18, Chicago, IL, USA). Continuous variables were expressed as mean ± standard deviation and were compared by performing Student’s t test. The Mann-Whitney rank sum test was used when the normality assumption of continuous data was not met. Categorical variables were compared using the chi-square test or Fisher’s exact test. Multiple stepwise binary logistic regression models were used to analyze the relationships of the clinical factors (age, GCS and NIHSS scores) as well as persistency of DWI lesions to functional outcomes. All statistical tests were 2-tailed and a *p* value of ≦0.05 indicated a significant statistical difference.

## Results

### Clinical factors associated with diffusion-restricted lesions

We prospectively enrolled 153 spontaneous ICH patients in this study during Jan. 2008 to Nov. 2013. The time from ICH onset to the initial MRI scan was 4.0±4.2 days. Among the 153 patients, 17 (11.1%) had one or more DWI lesions. The upper part of Table 1 summarizes demographics and clinical characteristics of the patients with and without DWI lesions. Patients with DWI lesions had higher systolic blood pressure (SBP) and mean arterial blood pressure (MAP) at the time they arrived at the ER (*P* = 0.049 and 0.043, respectively); a sharper decrease of the MAP between the time MAP was also recorded at the ER and the lowest MAP recorded during the first 24 hours after ICH (*P* = 0.030). Patients with positive DWI lesions also had a trend toward being older (*P* = 0.068), and having a more pronounced lowering of SBP and diastolic blood pressure (*P* = 0.072 and 0.052, respectively). There was no association between DWI lesions and conventional ICH risk factors. The GCS score at one week and functional outcomes at 3 months did not differ significantly between patients with and without DWI lesions.

### Markers of small vessel disease associated with diffusion-restricted lesions

The associations between DWI lesions and the four MRI markers of SVD are shown in the lower part of Table 1. More patients with DWI lesions had overall and deep (basal ganglia) WMH (*P* = 0.043 and <0.001, respectively) as well as overall, lobar, and deep CMB (*P* = 0.016, <0.001, and 0.003, respectively). The presence of EPVS and lacunae, regardless of their locations, were not associated with the presence of DWI lesions.

### Characteristics and fate of DWI lesions

The demographic and MRI data of the 17 patients with DWI lesions are shown in Table 2. The total number of DWI lesions was 25. A single lesion was seen in 11 patients (64.7%). Twenty DWI lesions were located in cortical or subcortical regions (80%); others were located in the basal ganglia, cerebellum, brain stem, or corpus callosum. Thirteen DWI lesions (52%) were not found on follow-up T2-weighted or FLAIR imaging. At the ER, the mean SBP and MAP of these 17 patients were 201.9±28.8 mmHg and 145.5±23.0 mmHg, respectively. The difference between the lowest MAP during the first 24 hours and MAP at the ER was 48.2±25.6 mmHg. Mixed periventricular and basal ganglia WMH was present in 16 patients (94.1%) while 1 patient had strictly basal ganglia WMH. Mixed centrum semiovale and basal ganglia EPVS was present in 13 patients (76.5%) while 4 patients had strictly basal ganglia WMH. Fourteen patients did not show old lacunae infarction on MRI (82.4%) while 2 patients had lacunae infarction in the cortical region and 1 patient had lacunae infarction in deep gray matter. Mixed lobar and deep CMB was noted in 11 patients (68.8%) while 1 patient had strictly lobar and another had strictly deep CMB.

**Table 2:** Demographic and MRI data for the 17 patients with DWI lesions.

No	Age	Gender	ICHlocation	Numberof DWIlesions	Location ofDWI lesion	Size of DWI lesion (cm)	Lesion onfollow-upMRI	7-day GCS	3-month mRS	SBP at ER(mmHg)	MAP at ER(mmHg)	△ MAP(mmHg)	WMH	EPVS	Lacunae	CMB
1	68	F	Cerebellum	1	Basal ganglia	0.8	Absent	15	2	196	131	45	Mixed	Mixed	Deep	nan
2	59	F	Putamen	2	Cortical;Subcortical	0.7; 0.6	Present;Absent	15	1	213	145	50	Mixed	Mixed	Absent	Absent
3	75	F	Putamen	1	Cerebellum	0.5	Present	15	4	183	120	10	Mixed	B.G	Absent	Lobar
4	85	F	Putamen	1	Cortical	0.4	Present	13	4	212	148	56	Mixed	Mixed	Absent	Mixed
5	45	F	Putamen	3	Cortical	2.7; 0.4; 0.4	Present;Absent;Absent	14	4	216	150	38	Mixed	B.G	Absent	Mixed
6	79	M	Thalamus	1	Cortical	0.5	Present	14	4	229	168	11	Mixed	Mixed	Cortical	Mixed
7	59	M	Putamen	2	Cortical	3.7; 0.7	Present;Present	6	4	185	128	28	Mixed	B.G	Absent	Mixed
8	76	M	Putamen	1	Basal ganglia	0.9	Absent	15	0	198	153	67	Mixed	Mixed	Absent	Mixed
9	73	M	Putamen	2	Cortical	0.7; 0.5	Present;Absent	15	4	157	135	37	Mixed	Mixed	Absent	Mixed
10	62	F	Putamen	1	Corpus callosum	3.4	Present	9	5	153	125	61	Mixed	B.G	Absent	Absent
11	79	F	Caudate	1	Subcortical	1.1	Present	6	3	240	167	77	Mixed	Mixed	Absent	Mixed
12	79	M	Putamen	1	Cortical	0.8	Absent	15	1	227	159	51	Mixed	Mixed	Cortical	Deep
13	68	F	Thalamus	2	Cortical	0.7; 0.7	Absent;Absent	15	0	232	165	72	Mixed	Mixed	Absent	Mixed
14	86	M	Thalamus	3	Cortical	1.3; 0.8; 0.7	Present;Absent;Absent	12	4	209	172	86	B.G	Mixed	Absent	Mixed
15	76	M	Putamen	1	Cortical	1.1	Absent	11	3	193	127	37	Mixed	Mixed	Absent	Mixed
16	63	F	Putamen	1	Brain stem	0.9	Present	9	5	151	95	5	Mixed	Mixed	Absent	Absent
17	44	M	Putamen	1	Cortical	1.2	Absent	11	4	238	185	88	Mixed	Mixed	Absent	Mixed

Abbreviations: n.a. = Not available; GCS = Glasgow Coma Scale; mRS = modified Rankin Scale; SBP = systolic blood pressure; WMH: white matter hyperintensity; MAP = mean arterial pressure; EPVS = enlarged perivascular spaces; CMB = cerebral microbleeds; B.G = basal ganglia.

Table 3 shows the correlations between the fate of DWI lesions with clinical factors, DWI lesion sizes and ADC values. DWI lesions that presented in follow-up T2WI/FLAIR imagings had lower ADC value (*P* = 0.003) and less lowering of MAP between the time MAP was recorded at the ER and the lowest MAP recorded during the first 24 hours after ICH (*P* = 0.033). Patients with any DWI lesions transiting into a final infarct (n = 11) had worse functional outcome (mRS = 3–6) compared to those without any persistent DWI lesion (n = 6) even after adjustment for age, initial GCS and initial NIHSS score (*P* = 0.028).

**Table 3:** Correlations between the fate of DWI lesions with clinical and imaging factors.

		DWI lesions in 3 month follow-up T2WI/FLAIR		
	All lesions (n = 25)	Present (n = 12)	Absent (n = 13)	*p*
Age, y	67.9 (13.5)	68.6 (12.6)	67.2 (14.8)	0.787
Time from stroke onset to MRI scan, day	4.3 (2.9)	5.3 (3.7)	3.3 (1.5)	0.101
DWI lesion size, cm	1.0 (0.8)	1.4 (1.2)	0.7 (0.2)	0.089
mean ADC value, 10^−6^ mm^2^/s	578.0 (104.9)	511.8 (112.9)	639.2 (43.6)	0.003*
SBP at ER, mmHg	200.1 (30.7)	194.4 (30.0)	205.4 (31.5)	0.383
MAP at ER, mmHg	146.3 (23.7)	140.1 (22.7)	151.9 (24.0)	0.222
Delta MAP (ER - lowest within 24 h), mmHg	51.3 (25.5)	40.7 (26.3)	62.0 (19.7)	0.033*

Results are expressed as mean (SD) for continuous variables.

Abbreviations: DWI = diffusion-weighted imaging; ADC = apparent diffusion coefficient; SBP = systolic blood pressure; DBP = diastolic blood pressure; MAP = mean arterial pressure; ER = emergency room.

*Significant differences were defined as those with *p*<0.05.

## Discussion

This prospective longitudinal MRI study of ICH confirms the findings of previous studies reporting a high incidence of new DWI lesions in acute ICH patients as well as the clinical and imaging factors that are associated with the development of DWI lesions including high and rapid lowering of blood pressure, and presence of WMH and CMB. Furthermore, we found that 52% of the DWI lesions were not visible on follow-up T2WI or FLAIR images. Though the presence of DWI lesions did not predict poor functional outcome at 3 months, transition of a DWI lesion into a final infarct was associated with poor functional outcome.

Two types of SVD, including hypertensive arteriopathy and cerebral amyloid angiopathy (CAA), have been proposed to be associated with deep and lobar ICH, respectively. As in previous studies we also found that the presence of two MRI markers for SVD, including WMH and CMB, are associated with new DWI lesions in ICH patients. [3], [4], [7], [9] A recent asymptomatic small DWI lesion was identified by chance on imaging, [12], [14] and has been referred to as silent cerebral infarction. In fact, a new DWI lesion has also been recognized as one of the neuroimaging markers for SVD. [15], [16] Although a new DWI lesion in SVD was defined as a recent small subcortical infarct of less than 20 mm in diameter, [12] a DWI lesion in ICH patients was commonly found in the cortex, ranging in incidence from 23.5% to 47%, [2], [3], [5], [7], [8] and sometimes with a diameter exceeding 20 mm. [4] In contrast to traditional subcortical infarcts in SVD, the cortical DWI lesions in ICH patients may have different underlying pathophysiology, such as CAA, rather than hypertensive arteriopathy alone.

The incidence of DWI lesions in ICH patients in our study was 11.1%, which is relatively low compared with previous reports (13–41%). [2]–[7] In our patients, most hematomas (91.5%) were located in deep brain regions. Of the 74 patients who presented with CMB, 69 (93.2%) had CMB in a deep location. This indicates that the etiology of ICH in our patients was mostly hypertensive arteriopathy (the major cause of ICH in Asia) instead of CAA. Although most of our patients had deeply located CMB, 12 out of 17 patients with DWI lesions had lobar or mixed lobar and deep CMBs, which is noteworthy insofar as lobar CMB has been recognized as a marker of CAA. [3], [17] It is possible that hypertensive arteriopathy and CAA co-existed in most patients with DWI lesions in our study. In a paper by Gregoire and colleagues, [3] it was reported that the prevalence of DWI lesions was 23% in patients with probable CAA-related ICH versus 8% in the remaining ICH patients, and there was a strong association between DWI lesion and lobar CMB. In the Kimberly et al. study, [8] 15% of the subjects with a diagnosis of probable CAA had a DWI lesion on MRI. CAA is characterized by the progressive accumulation of amyloid-β in the walls of small arteries and arterioles predominantly located in the leptomeningeal space and cortex. [18] These changes not only can cause the vessel wall to become disrupted and fragmented, and prone to blood extravasation and microaneurysm formation, but also can cause dysfunction of blood flow regulation and luminal occlusion. [19]–[21] Another possible explanation is that the presence of a DWI lesion may represent more severe hypertensive vasculopathy that can leads to more CMBs in both deep and lobar regions.

Previous research has shown that DWI lesions are associated with significant decline of blood pressure within 24 hours, which was confirmed by our study. [2], [4], [6] The mechanism for greater decline of blood pressure in ICH patients with DWI lesions is not yet well understood. SVD is sufficient to cause cerebral ischemia, especially after ICH when there is an increase of intracranial pressure (ICP) and an acute trigger of prothrombotic/proinflammatory cascades. The frequency of ischemia is even increased when SVD and increased ICP are accompanied by a sudden dramatic lowering of blood pressure. This “triple-hit” hypothesis may explain the high incidence of DWI lesions in ICH patients, especially when there is acute reduction of blood pressure.

The signal increases on DWI most likely result from a reduction in the Brownian motion of water molecules. Once the sodium potassium pump fails, the water molecules shift from the extracellular to intracellular space and thus reduce diffusivity. This has been widely regarded as cytotoxic edema, which indicates irreversible tissue damage. [22], [23] However, both animal and human studies have demonstrated that DWI lesions can be reversible. [24], [25] Positron emission tomography studies have revealed that the degree of metabolic disruption within the DWI lesion is variable, and DWI lesions may not always represent irreversible tissue injury. [15], [26] A systemic review revealed that partial DWI lesion reversal occurred in 24% of patients within a few days after stroke. [16] Some studies have shown that DWI lesions might have different fates, evolving to visible, long-term small lacunae or to white matter lesions on T2-weighted imaging, or might disappear with time. [12] Our results show that 52% of DWI lesions in ICH patients were not found on follow-up T2-weighted images or FLAIR images, and that all of the DWI lesions were subclinical. The regression of DWI lesions was associated with higher ADC value, which may be associated with more reversible vasogenic edema rather than irreversible cytotoxic edema. [27] Further, regression of DWI lesions was also associated with a sharper decrease in MAP during the first 24 hours. For this reason, the transient restriction of diffusion after ICH may be due to a sudden lowering of blood pressure. Taken together, a DWI lesion in acute ICH patients should be regarded as another marker of SVD or as silent ischemia; it does not always indicate a cerebral infarct or permanent tissue injury.

Whether the presence of a DWI lesion can predict poor functional outcome is still under debate. In a study by Garg and colleagues, [6] DWI lesions were found in multivariate analysis to be associated with greater decline of blood pressure and with disability and death at 3 months. The benefits of current blood pressure management in ICH patients to reduce hematoma volume and improve functional outcome are thus thought to be limited by DWI lesions. [4], [6], [9] However, in a Kang et al. study, [5] as well as in our study, DWI lesions were not related to functional outcome at 3 months. Interestingly, patients with good functional outcome in our study had more DWI lesions that were not visible on follow-up T2WI or FLAIR images. It is hypothesized that a DWI lesion is a marker of SVD, whereas regression of a DWI lesion indicates a minor vasculopathy and a transient vascular occlusion with early reperfusion. Further studies, however, are needed to determine the natural course of DWI lesions and to identify how these lesions are affected by management strategies, and how they are related to functional outcome.

This study had several limitations. The time interval of a baseline MRI scan ranged from 1 to 14 days after ICH, and it is possible that some DWI lesions were present in the early acute stage and had reversed by the time of the MRI scan. It is also possible that some patients who were scanned early developed DWI lesions later in the subacute stage. These possibilities may have led to an underestimation of the true incidence of DWI lesions. Also, patients with contraindications or co-morbidities that were unstable for MRI were excluded, thus resulting in selection bias. Finally, the number of patients with DWI lesions was low and more cases are necessary to further survey the risks and clinical impacts of DWI lesions.

## Conclusions

In conclusion, this study confirms that DWI lesions in ICH patients are associated with CMB and WMH as well as acute reduction of blood pressure. Presence of a DWI lesion may be due to underlying amyloid angiopathy or a more severe hypertensive vasculopathy. More than half of the DWI lesions in our ICH patients did not result in observable, long-term infarcts. Only DWI lesions transforming finally into infarcts predicted poor functional outcome. A DWI lesion may be regarded as a marker of SVD or as silent ischemia, and does not always indicate an absolute cerebral infarct or permanent tissue injury. Further studies should clarify how management strategies, including blood pressure control, early hydration, and antithrombotic and osmotic agents, will affect the presence and regression of DWI lesions.
